# Transcriptome analysis of the common moss *Bryum pseudotriquetrum* grown under Antarctic field condition

**DOI:** 10.1093/aobpla/plae043

**Published:** 2024-08-10

**Authors:** Masahiro Otani, Haruki Kitamura, Sakae Kudoh, Satoshi Imura, Masaru Nakano

**Affiliations:** Faculty of Agriculture, Niigata University, 2-8050 Ikarashi, Nishi-ku, Niigata 950-2181, Japan; Graduate School of Science and Technology, Niigata University, 2-8050 Ikarashi, Nishi-ku, Niigata 950-2181, Japan; National Institute of Polar Research, Research Organization of Information and Systems, 10-3 Midori-cho, Tachikawa-shi, Tokyo 190-8518, Japan; Polar Science, SOKENDAI (The Graduate University for Advanced Studies), Hayama, Kanagawa 240-0193, Japan; National Institute of Polar Research, Research Organization of Information and Systems, 10-3 Midori-cho, Tachikawa-shi, Tokyo 190-8518, Japan; Polar Science, SOKENDAI (The Graduate University for Advanced Studies), Hayama, Kanagawa 240-0193, Japan; Faculty of Agriculture, Niigata University, 2-8050 Ikarashi, Nishi-ku, Niigata 950-2181, Japan

**Keywords:** Antarctic moss, environmental stress, environmental adaptation, fatty acid, lipid metabolism

## Abstract

Mosses are distributed all over the world including Antarctica. Although Antarctic mosses show active growth in a short summer season under harsh environments such as low temperature, drought and high levels of UV radiation, survival mechanisms for such multiple environmental stresses of Antarctic mosses have not yet been clarified. In the present study, transcriptome analyses were performed using one of the common mosses *Bryum pseudotriquetrum* grown under an Antarctic field and artificial cultivation conditions. Totally 88 205 contigs were generated by *de novo* assembly, among which 1377 and 435 genes were significantly up and downregulated, respectively, under Antarctic field conditions compared with artificial cultivation conditions at 15°C. Among the upregulated genes, a number of lipid metabolism-related and oil body formation-related genes were identified. Expression levels of these genes were increased by artificial environmental stress treatments such as low temperature, salt and osmic stress treatments. Consistent with these results, *B*. *pseudotriquetrum* grown under Antarctic field conditions contained large amounts of fatty acids, especially α-linolenic acid, linolenic acid and arachidonic acid. In addition, proportion of unsaturated fatty acids, which enhance membrane fluidity, to the total fatty acids was also higher in *B*. *pseudotriquetrum* grown under Antarctic field conditions. Since lipid accumulation and unsaturation of fatty acids are generally important factors for the acquisition of various environmental stress tolerance in plants, these intracellular physiological and metabolic changes may be responsible for the survival of *B*. *pseudotriquetrum* under Antarctic harsh environments.

## Teaser

Antarctica has harsh environments including low temperatures, drought and high levels of UV radiation etc. Although several moss species grow in Antarctica, their survival and adaptation mechanisms for such multiple environmental stresses have not yet been known. Here, we performed transcriptome analyses using one of the common mosses *B. pseudotriquetrum* grown in Antarctica. Our results showed a possibility that lipid accumulation and change in fatty acid composition are major mechanisms for acquiring environmental stress tolerance in Antarctic mosses. In addition, we proposed the importance of on-site analysis for elucidating actual plant environmental responses and stress tolerance mechanisms in nature.

## Introduction

In ice-free areas of Antarctica, limited organisms live under harsh environments such as low temperatures, drought and high UV radiation. Among these organisms, several moss species grow as dominant terrestrial plants. Therefore, these mosses are assumed to have high adaptability to harsh environments. However, little is known about the molecular mechanisms of environmental stress tolerance of mosses grown in Antarctica.

When plants are exposed to severe environments, stress response genes, such as myelocytomatosis (MYC) genes, myeloblastosis (MYB) genes, ethylene-responsive element binding factor (ERF) genes and dehydration-responsive element binding (DREB) genes, are induced, and subsequently various stress responses including expression of molecular chaperon proteins, production of antioxidants such as flavonoids, accumulation of sugars and lipids (Lata and Prasad 2011; He *et al*. 2018; Hrmova and Hussain 2021). Lipid accumulation and changes in fatty acid composition are one of the most important mechanisms, especially for cold stress tolerance in plants (Barrero-Sicilia *et al*. 2017; Reszczyńska and Hanaka 2020). The fluidity of cell membranes increases with the proportion of unsaturated fatty acids in the membrane phospholipids, and plants maintain cell membrane function by increasing the proportion of unsaturated fatty acids to increase membrane fluidity under cold stress conditions. Also, triacylglycerol (TAG), which is biosynthesized from free fatty acids in the endoplasmic reticulum, is accumulated in the oil body for acclimatization to low temperatures (Degenkolbe *et al*. 2012). In some moss species, unsaturated fatty acids are accumulated under low-temperature conditions as in seed plants (Aro and Karunen 1988; Beike *et al*. 2014, 2015; Resemann *et al*. 2021; Lu *et al*. 2022). Thus, modification of lipid metabolism for cold acclimatization and acquisition of cold stress tolerance seems to be a common system in plants.

In *Pohlia nutans* (Hedw.) Lindb., one of the mosses grown in Antarctica, changes in gene expression profiles have been investigated in response to artificial stress treatments such as high salinity, low temperature and drought (Zhang *et al*. 2019; Liu *et al*. 2021, 2022; Fang *et al*. 2022). In these studies, expression of transcription factor genes involved in environmental stress response and tolerance (e.g. MYC, MYB, ERF and DREB genes), genes involved in signalling of plant hormones such as abscisic acid (ABA) and jasmonic acid, and antioxidant biosynthesis genes have been shown to be induced by artificial stress treatment. However, no studies using mosses grown under Antarctic field conditions have so far been reported, and gene expression profiles of mosses grown under natural Antarctic conditions are still unclear. The common moss *Bryum pseudotriquetrum* (Hedw.) P. Gaertn., B. Mey. and Scherb. is distributed throughout the world and is one of the dominant moss species in Antarctica. Therefore, this moss is expected to have a high tolerance to environmental stresses. In the present study, transcriptome analysis was performed to reveal gene expression profiles in *B*. *pseudotriquetrum* grown in Antarctica. This is the first report on gene expression profiling of mosses grown under Antarctic field conditions and provides important information for understanding the adaptation and tolerance mechanisms of mosses to Antarctic harsh environments.

## Materials and Methods

### Sampling of mosses

Blocks of *Bryum pseudotriquetrum* were cut out and collected from moss colonies at four different spots of ice-free regions of Langhovde, Skarvsnes and Skallen in east Antarctica during the summer season (from 29 December 2018 to 17 January 2019) (Fig. S1). GPS location data and environmental conditions of each spot are shown in Supporting Information Table S1. After collection, moss blocks were divided into several pieces (~2 cm squares) and some of them were immediately fixed in Farmer’s solution (acetic acid:ethanol = 1:3, v:v), stored at –20°C and used for RNA extraction. The rest of the moss blocks were stored at –20°C without fixation and used for artificial cultivation and LC–MS/MS analysis of fatty acids. Mosses, with or without fixation, directly used for analysis were defined as field samples (FSs).

### Artificial cultivation of mosses

After 6 months from collection, stored moss blocks collected from each spot without fixation were slowly thawed at 4 °C. They were then cultivated separately on vermiculite with liquid BCD medium (Nishiyama *et al*. 2000) under continuous illumination with light emitting diode lighting (200 μmol m^–2^ s^–1^) at 15 °C. After several weeks of incubation, green gametophores appeared on moss blocks. Such artificially cultivated mosses were defined as cultivated samples (CSs). Before RNA extraction, gametophores from CSs were fixed in Farmer’s solution and stored at –20 °C for two weeks in order to eliminate the effects of fixation on gene expression profiles.

### RNA extraction and construction of cDNA libraries for RNA-seq analysis

The flow of RNA extraction and RNA-seq analysis is shown in Supporting Information Fig. S2. Approximately 100 mg fresh weight (FW) of gametophores were used for RNA extraction. Total RNAs were extracted using Trizol regent (Thermo Fisher Scientific, MA, USA), and then purified by RNeasy Mini kit (QIAGEN, Hilden, Germany) and RNase-Free DNase Set (QIAGEN, Hilden, Germany) according to the manufacturer’s instructions. Extracted RNAs were further purified by ethanol precipitation. For FSs, RNA extraction was separately performed from gametophores of different blocks derived from the same moss colony and these samples were used as biological replicates. Then, total RNAs extracted from mosses collected at four different spots were mixed by an equal amount and used for cDNA library construction. Likewise, for CS, RNA extraction was performed three times separately from gametophores of cultured mosses derived from the same four spots was used. RIN values of total RNAs from CSs and FSs were 7.5–7.7 and 2.3–2.6, respectively. rRNA was depleted using RiboMinus^™^ Plant Kit for RNA-Seq (Thermo Fisher Scientific, MA, USA). cDNA libraries were constructed using the NEBNext Ultra II Directional RNA Library Prep Kit for Illumina (New England Biolabs Inc., MA, USA) according to the manufacturer’s instructions. Since total RNAs from FSs were partially fragmented, the fragmentation step of library construction was omitted. cDNA libraries were multiplexed at equal proportions (2 nm) and loaded onto a single flow cell of NextSeq500/550 Mid Output Kit v2.5 (150 cycles) (Illumina, CA, USA). Sequencing was performed on NextSeq 500 (Illumina, CA, USA) with three biological replicates for each sample.

### *De novo* assembly, differential expression analysis, and annotation

Low quality reads and contaminated reads derived from rRNAs were filtered from raw transcriptome sequences using FastqPuri version 1.0.7 (Pérez-Rubio *et al*. 2019) with the parameter -q 30 (minimum quality score: 30) and SortMeRNA version 4.2.0 (Kopylova *et al*. 2012). Filtered reads were assembled using TransLiG version 1.3 (Liu *et al*. 2019) with default parameters. Contaminated contigs derived from other organisms such as fungi and bacteria were detected by EnTAP version 0.9.2 (Hart *et al*. 2020) and removed. Transcripts Per Million value of transcripts was quantified by Salmon version 1.3.0 (Patro *et al*. 2017) and differential expressed genes (DEGs) were generated using tximport version 1.12.3 (Soneson *et al*. 2015) and DESeq2 version 1.24.0 R package (Love *et al*. 2014). Gene ontology (GO) annotation of DEGs was performed using EnTAP version 0.9.2.

### Cold, salt, and drought stress treatments

Axenic cultures of mosses collected at spot 4 were used for artificial stress treatments. Gametophores cultivated on vermiculite were surface-sterilized with a sodium hypochlorite solution containing 1 % active chlorine and cultured on a BCD solid medium with 0.8 % agar under continuous illumination at 15 °C. After several months, fully elongated gametophores were isolated from proliferated moss cultures, transferred to fresh solid BCD medium, and cultured for 3 days in order to avoid the effect of gametophore cutting on gene expression. For cold stress treatment, gametophores were further transferred to fresh BCD solid medium and cultured under a long-day condition (16 h light, 8 h dark) at 4 °C. For salt and drought stress treatments, gametophores were further transferred to BCD solid media containing 200 mm NaCl and 300 mm mannitol, respectively, and cultured under continuous illumination at 15 °C. Each stress treatment was performed three times separately and these samples were used as three biological replicates. For each treatment, gametophores were collected weekly and used for gene expression analysis.

### Real-time RT–PCR analysis

Extraction of total RNAs from gametophores and cDNA synthesis were performed using Plant Total RNA Purification Kit (BioElegen Technology Co., Ltd., Taichung, Taiwan) and ReverTra Ace^®^ qPCR RT Master Mix (Toyobo Co., Ltd., Osaka, Japan) according to the manufacturer’s instructions. Real-time RT–PCR was performed using KOD SYBR^®^ qPCR Mix (Toyobo Co., Ltd., Osaka, Japan) on the MyGo PCR systems (IT-IS Life Science Ltd., Dublin, Ireland). The specificity of the amplification product was confirmed by melting curve analysis. Real-time RT–PCR analysis was performed in biological triplicate for each sample. Relative amounts of transcripts were calculated using the comparative cycle threshold method (Livak and Schmittgen 2001), and results were normalized to BTB/POZ domain-containing protein gene of *B*. *pseudotriquetrum* (*BpPOB1*; LC767358 from the GenBank/EMBL/DDBJ databases). We have confirmed that there are no significant differences in expression levels of *BpPOB1* between FS and CS. Primer sets used are listed in see Supporting Information Table S2.

### LC–MS/MS analysis of fatty acids

Gametophores from FSs and CSs, which were corrected at spots 1 and 4, were used for fatty acid analysis (see Supporting Information Fig. S1). Twenty milligram FW of samples were homogenized in liquid nitrogen, and fatty acids were extracted in chloroform/methanol (50:50, v/v). Purified extracts were then analysed using Prominence XR (Shimadzu, Kyoto, Japan) attached to LTQ Orbitrap XL (Thermo Fisher Scientific, MA, USA) with *L-column3 ODS* (2.1 mm I.D. × 150 mm with 2 μm internal diameter, CERI, Tokyo, Japan). Fatty acid concentrations were calculated from peak areas using MRMPROBS ver. 2.60 software. Sixty-seven fatty acids were initially analysed using FSs, and the top 15 fatty acids with higher contents were further investigated. Although each analysis was performed in a single replication, data reliability was ensured by examining samples from two different sampling spots.

## Results

### RNA-seq analysis using FSs and CSs

**Figure 1.**
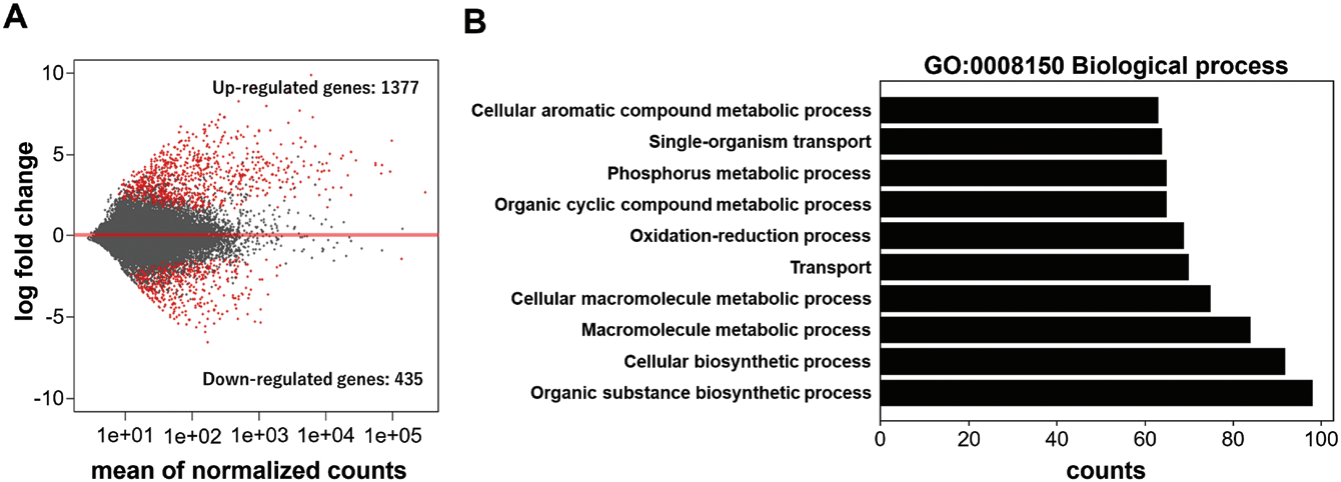
Identification of differentially expressed genes (DEGs) and gene ontology (GO) enrichment analysis of DEGs. (A) MA plot of DEGs. Each dot represents a single gene. Red dots indicate significantly up or downregulated genes in FSs compared with CSs (q-value < 0.01). (B) GO enrichment analysis of upregulated DEGs using the GO term biological process (GO:0008150).

In order to examine up and downregulated genes in FSs compared with CSs, *de novo* assembly and identification of differentially expressed genes (DEGs) were carried out using transcriptome data. In our transcriptome data, although the number of total reads were low (3–4.7 million), the mapping rates indicating the reliability of the data were good (CSs: ~91.7–92.9%, FSs: ~77.6–86.2%; see Supporting information Table S3). Totally 88 205 contigs were generated by *de novo* assembly (see Supporting information Table S4). Among them, 1377 and 435 genes were significantly up and downregulated, respectively, under Antarctic field conditions (Fig. 1A). GO enrichment analysis showed that upregulated DEGs contained many genes involved in various biosynthetic and metabolic processes, such as cellular macromolecule metabolic process, macromolecule metabolic process, cellular biosynthetic process and organic substance biosynthetic process (Fig 1B). A number of genes involved in lipid metabolism and accumulation were also found in the upregulated DEGs (Table 1). Especially, genes for oleosin Bn-III, which are localized in the oil drop membrane and involved in oil body formation, Δ-15 fatty acid desaturase (Δ15FAD; FAD3), which involved in the conversion from linoleic acid (LA) to α-linolenic acid (ALA) in ERs, and LPTs, which is involved in the transport of wax to cuticular layer; were highly expressed under Antarctic field conditions (Table 1; Fig. 3). On the other hand, downregulated DEGs contained many photosynthesis-related genes (see Supporting Information Fig. S3). In particular, expression levels of most genes encoding proteins constituting light-harvesting chlorophyll protein complex (LHC) were downregulated.

**Table 1.** Upregulated DEGs involved in lipid metabolism and oil body formation in FSs.

Gene ID	log2_Fold_Change	padj	Results of BLAST search
Graph_942	3.247785	1.95E – 78	Oleosin Bn-III
Graph_1034	2.307131	2.01E – 48	Rubber elongation factor/small rubber particle protein (REF/SRPP)
Graph_8732	4.032544	1.42E – 13	Delta-15 fatty acid desaturase (Δ15FAD; FAD3)
Graph_1415	2.714195	3.44E – 10	Delta-15 fatty acid desaturase (Δ15FAD; FAD3)
Graph_3920	1.935774	1.01E – 08	Delta-12 fatty acid desaturase, endoplasmic reticulum (Δ12FAD; FAD2)
Graph_1605	1.707515	1.80E – 13	Delta-12 fatty acid desaturase, chloroplast (Δ12FAD; FAD6)
4287	3.57154	2.19E – 04	Delta-8 fatty acid desaturase (Δ8FAD; FADS2)
6000	2.346697	0.001842	Lysophospholipid acyltransferase 1 (LPCAT1)
Graph_4090	1.293359	2.41E – 05	Phosphatidate cytidylyltransferase 1 (CDS1)
Graph_1342	2.596322	2.06E – 40	Linoleate 9S-lipoxygenase (9-LOX)
Graph_3502	1.065160	2.91E – 05	Cytochrome b5
Graph_9286	1.556220	3.94E – 05	Nonspecific lipid transfer protein (ns-LTP)
Graph_3730	5.508316	5.48E – 23	Nonspecific lipid-transfer protein 2B (ns-LTP 2B)

Expression levels of plant core environmental stress response (PCESR) genes, a gene set commonly induced by various environmental stresses in plants (Hahn *et al*. 2013), were examined. Although 54 out of the 56 PCESR genes were detected in the transcriptome data, only seven showed elevated expression levels in FSs (see Supporting information Table S4).

### Expression analysis of oil body formation-, lipid metabolism- and lipid transfer-related genes

**Figure 2.**
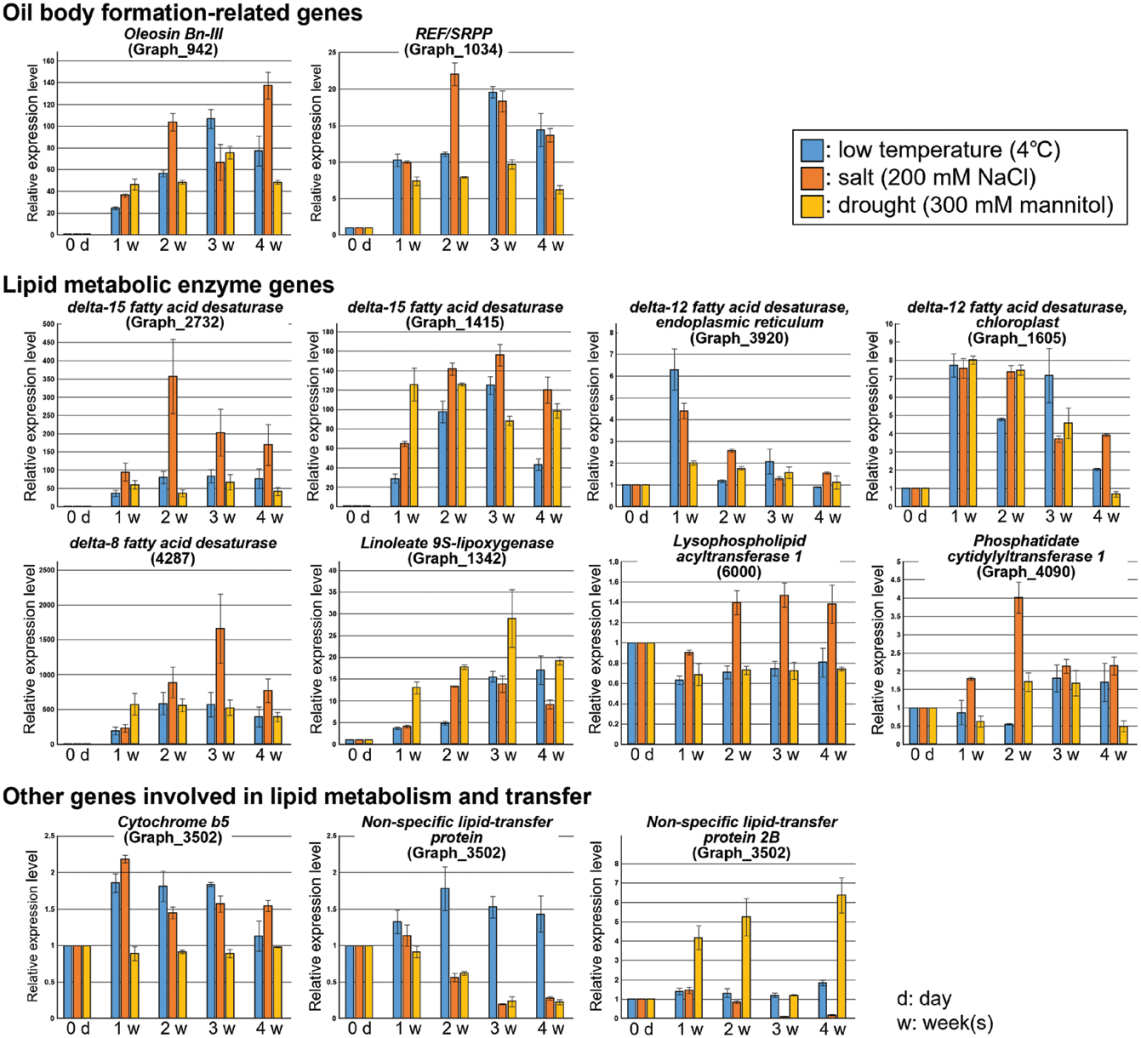
Real-time RT–PCR analysis of lipid metabolism-related and oil body formation-related genes. Low-temperature (4 °C), high-salt (200 mm NaCl), or drought (300 mm mannitol) stress treatments were applied to the gametophore of *Bryum pseudotriquetrum* for 1–4 weeks. Relative amounts of transcripts of each gene were normalized to *BpPOB1*. Values represent the means ± standard error of triplicates.

In order to investigate the response of oil body formation-, lipid metabolism- and lipid transfer-related genes to various environmental stresses, expression analysis of these genes was carried out in CSs treated with low temperature, salt and drought stresses (Fig. 2). Genes for oleosin Bn-III and rubber elongation factor/small rubber particle protein (REF/SRPP) involved in oil body formation responded to all stress treatments. Expression levels of these genes increased up to 106.6- and 19.6-fold by cold stress treatment, 137.1- and 22.0-fold by salt stress treatment, and 67.5- and 9.7-fold by drought stress treatment within 4 weeks, respectively. Likewise, expression levels of lipid metabolism-related genes, *Δ12FAD* (*FAD2/6*), *Δ15FAD*, *Δ8FAD* and *9-LOX* genes were upregulated in response to all stress treatments. In particular, expression levels of *Δ15FAD* and *Δ8FAD* greatly increased up to 127.7- and 583.5-fold by cold stress treatment, 356.8- and 1660.2-fold by salt stress treatment, and 125.8- and 572.6-fold by drought stress treatment within 4 weeks, respectively. Expression levels of *LPCAT1*, *CDS1*, *cytochrome B5* and *NsLTPs* increased in response to at least one of the stress treatments.

### Identification and quantification of fatty acids by LC–MS/MS analysis

Fatty acid composition and content in FSs collected at different two spots, Langhovde (spot 1) and Skallen (spot 4), and CSs collected at the same spots were investigated (Table 2). Both FSs contained mainly α-linolenic acid and linolenic acid as major fatty acids, followed by arachidonic acid, palmitic acid, oleic acid, γ-linolenic acid, dihomo-γ-linolenic acid, EPA and cis-vaccenic acid. The total amount of fatty acids was greatly higher in FSs compared to CSs. The content of a major saturated fatty acid, palmitic acid (C16:0), was 5.8- to 8.3-fold higher in FSs, while contents of two major unsaturated fatty acids, α-linolenic acid (C18:3*n* – 3) and linolenic acid (C18:2*n* – 6), were 27.9- to 30.8-fold and 33.3- to 138.5-fold higher in FSs, respectively. Thus, the proportion of unsaturated fatty acid contents among total fatty acid contents was much higher in FSs.

**Table 2.** Amount of fatty acids in gametophores of FSs and CSs (μg/g).

Fatty acids		*B*. *pseudotriquetrum* collected in sampling spot 1		*B*. *pseudotriquetrum* collected in sampling spot 4	
		FS	CS	FS	CS
α-Linolenic acid	C18:3*n* – 3	39 000	1400	37 000	1200
Linolenic acid	C18:2*n* – 6	30 000	900	15 000	390
Arachidonic acid	C20:4*n* – 6	9800	1300	8400	830
Palmitic acid	C16:0	6900	1200	7900	950
Oleic acid	C18:1*n* – 9	2700	61	3000	45
γ-Linolenic acid	C18-3*n* – 6	6200	140	2900	87
Dihomo-γ-Linolenic acid	C20:3*n* – 6	3700	70	1900	21
Eicosapentaenoic acid	C20:5*n* – 3	1500	180	1600	130
cis-Vaccenic acid	C18:1*n* – 7	1500	60	1100	69
Palmitoleic acid	C16:1*n* – 7	160	21	400	25
8Z, 11Z, 14Z, 17Z-Eicosatetraenoic acid	C20:4*n* – 3	490	10	320	8
Nervonic acid	C24:1*n* – 9	550	28	320	18
11Z, 14Z-Eicosadienoic acid	C20:2*n* – 6	360	13	310	6
Stearidonic acid	C18:4*n* – 3	630	8	290	6
11Z, 14Z, 17Z-Eicosatrienoic acid	C20:3*n* – 3	160	15	190	12

## Discussion

**Figure 3.**
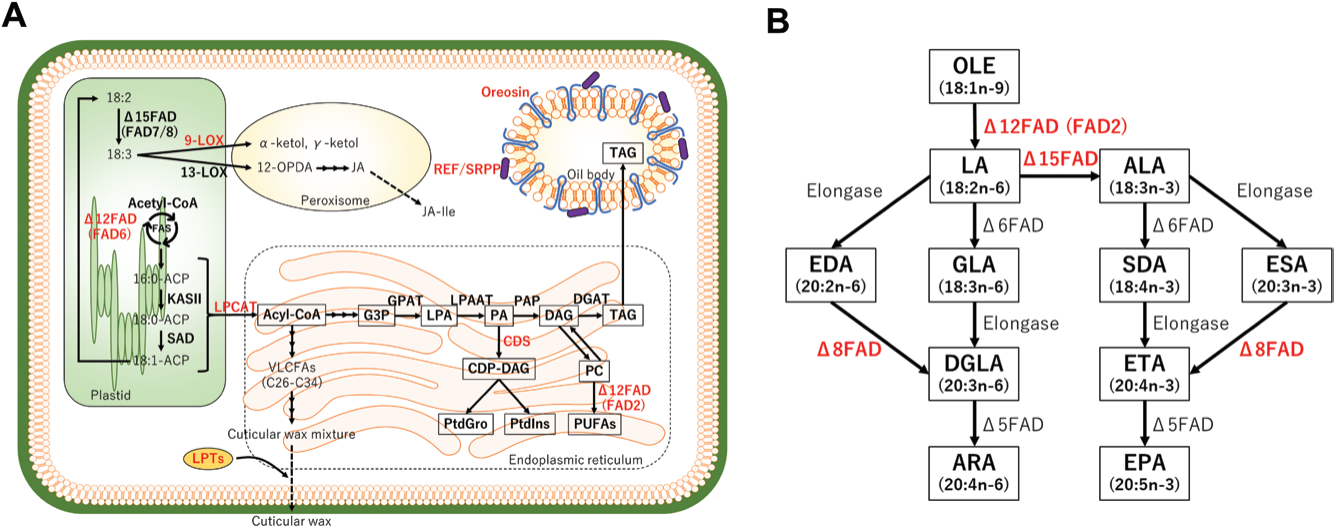
Overview of lipid metabolic pathways in plants (A) and major desaturation and elongation steps of polyunsaturated fatty acids in Endoplasmic reticulum (B). Enzymes whose expression are upregulated in FSs are indicated by red letters. Abbreviations are as follows: 10-OPDA, 10-Oxo-11,15-phytodienoic acid; 12-OPDA, 12-Oxo-10,15-phytodienoic acid; ACP, acyl carrier protein; ALA, α-Linolenic acid; ARA, Arachidonic acid; CDP-DAG, cytidine diphosphate diacylglycerol; CDS, CDP-diacylglycerol synthase; CoA, coenzyme A; DAG, diacylglycerol; DGAT, diacylglycerol acyltransferase; DGLA, dihomo-γ-linolenic acid; EDA, eicosadienoic acid; EPA, eicosapentaenoic acid; ESA, eicosatrienoic acid; ETA, eicosatetraenoic acid; FAD, fatty acid desaturase; FAD, fatty acid desaturase; FAS, type II fatty acid synthase complex; G3P, glycerol-3-phosphate; GLA, γ-Linolenic acid; GPAT, glycerol-3-phosphate acyltransferase; JA, jasmonic acid; JA-Ile, jasmonic acid-isoleucine; KAS, β-ketoacyl-ACP synthase; LA, linoleic acid; LOX, lipoxygenase; LPA, lysophosphatidic acid; LPAAT, lysophosphatidic acid acyltransferase; LPCAT, lysophosphatidylcholine acyltransferase; LPTs, lipid transfer proteins; OLE, oleic acid; PA, phosphatidic acid; PAP, phosphatidic acid phosphatase; PC, phosphatidylcholine; PtdGro, phosphatidylglycerol; PtdIns, Phosphatidylinositol; PUFAs, polyunsaturated fatty acids; REF/SRPP, rubber elongation factor/small rubber particle protein; SAD, Δ9 stearoyl-acyl carrier protein desaturase; SDA, stearidonic acid; TAG, triacylglycerol; VLCFAs, very long chain fatty acids..

In the present study, RNA-seq analysis was carried out in the common moss *B*. *pseudotriquetrum* and genes related to lipid metabolism and oil body formation were found to be highly expressed in FSs (Table 1; Fig. 3). In plant cells, lipid accumulation and changes in fatty acid composition are important mechanisms for acquiring environmental stress tolerance. Thus, these genes may be involved in multiple stress tolerance in *B*. *pseudotriquetrum* growing in Antarctica (Fig. 2).

Oil body membrane proteins such as oleosin and REF/SRP are involved in the regulation of the number and size of oil bodies and the accumulation of lipids in cells (Tzen *et al*. 1993; Ting *et al*. 1996). In *Arabidopsis thaliana* and *Oryza sativa*, lipid contents in leaves or seeds were increased by overexpression of the oleosin gene (Fan *et al*. 2013; Liu *et al*. 2013). Likewise, lipid accumulation was promoted by co-overexpression of the oleosin gene and genes involved in TAG-biosynthesis (Bhatla *et al*. 2010; Vanhercke *et al*. 2014). Oil body membrane proteins are also involved in the acquisition of environmental stress tolerance through oil body formation. In seeds of *A*. *thaliana*, oleosin has been shown to contribute to freezing tolerance by preventing abnormal fusion of oil bodies (Shimada *et al*. 2008). Overexpression of the REF/SRPP gene increased drought stress tolerance, whereas mutation or RNAi suppression of the REF/SRPP gene reduced drought tolerance in *A*. *thaliana* (Kim *et al*. 2016; Laibach *et al*. 2018). In the present study, oleosin and REF/SRP genes were highly expressed and large amounts of fatty acids were accumulated in FSs (Tables 1 and 2). Therefore, oil body membrane proteins may be involved in environmental stress tolerance through the promotion of oil body formation and lipid accumulation in cells of *B*. *pseudotriquetrum* grown under Antarctic field conditions.

An increase in membrane fluidity is essential for reducing the damage of environmental stresses such as low temperatures to cell membrane function. Generally, higher proportions of unsaturated fatty acids to the total fatty acids increase the fluidity of the cell membrane (Mikami and Murata 2003). Unsaturated fatty acids are produced through elongation reactions and unsaturation by various elongases and FADs. Cytochrome b5 acts as an electron donor to FADs in this process. In *Solanum lycopersicum*, *LeFAD3* (Δ15FAD gene of *S*. *lycopersicum*) expression was upregulated by a cold stress treatment, and overexpression of *LeFAD3* increased α-linolenic acids (C18:3*n* – 3) contents and induced cold stress tolerance (Yu *et al*. 2009). FAD2 is essential for proper function of the membrane-attached Na^+^/H^+^ antiporter and maintenance of low cytosolic Na^+^ levels, and *A*. *thaliana* fad2 mutant (mutant for endoplasmic reticulum-localized Δ12FAD) showed high sensitivity to salt stress (Zhang *et al.* 2012). In addition, the melting point of fatty acids decreases as the degree of their unsaturation increases, e.g. 13.4°C for oleic acid (18:1*n* – 9, mono-unsaturated fatty acid), –5°C for linoleic acid (18:2*n* – 6, di-unsaturated fatty acid), –11°C for α-linolenic acid (18:3*n* – 3, tri-unsaturated fatty acid), and –49.5°C for arachidonic acid (20:4*n* – 6, tetra-unsaturated fatty acid). In the present study, some FAD genes were highly expressed and unsaturated fatty acids were mainly accumulated in FSs (Tables 1 and 2; Fig. 3). Therefore, the unsaturation of lipids and the change of fatty acid composition may be important adaptation mechanisms to harsh environments in Antarctic mosses.

Very long-chain fatty acids (VLCFAs; C26–C36) are substrates of a cuticle wax mixture covering the plant surface, and therefore, fatty acids are also involved in physical protection against environmental stresses. Lipid transfer proteins (LTPs) are involved in the transport of a cuticular wax mixture to the cuticular layer. In many plant species, it has been reported that the expression of LPT genes is upregulated in response to abiotic/biotic stresses and contributes to multiple stress tolerance (Guo *et al*. 2013; Gangadhar *et al*. 2016; Martín-Pedraza *et al*. 2016; Xu *et al*. 2018). Among LTP family proteins, non-specific LTPs (nsLTPs) are also involved in cuticle formation, wax biosynthesis, plant growth and development (Sterk *et al*. 1991; Carvalho and Gomes 2007). In *Nicotiana glauca*, the expression level of a nsLTP gene and cuticular wax accumulation increased in response to drought stress, resulting in increased drought resistance (Cameron *et al*. 2006). In the present study, some nsLPT genes were highly expressed in FSs (Table 1). Thus, physical protection may also be enhanced by the reinforcement of extracellular matrix in mosses growing under Antarctic environments.

Expression of many of genes involved in lipid metabolism and accumulation, such as FADs and oleosin, increased after relatively long periods of various artificial stress treatments (>1 week) (Fig. 2). This result indicates that the acquisition of environmental stress tolerance in Antarctic mosses by changing fatty acid metabolism requires relatively prolonged exposure to environmental stresses. Although most previous studies using Antarctic mosses have examined changes in gene expression profiles after single artificial stress treatments for short periods (a few hours to several days) (Zhang *et al*. 2019; Liu *et al*. 2021, 2022; Fang *et al*. 2022), longer periods of stress treatments may be required to clarify actual mechanisms of environmental stress tolerance in Antarctic mosses.

In the present study, expression levels of photosynthesis-related genes, especially light-harvesting chlorophyll (LHC) protein genes, decreased in FSs grown under a unique daylength condition in Antarctic summer (midnight sun). It has been reported that LHC proteins are involved both in collecting light energy for driving the primary photochemical reactions of photosynthesis and in photoprotection when absorbed light energy exceeds the capacity of the photosynthetic apparatus (Rochaix and Bassi 2019). In *A*. *thaliana*, expression levels of several LHC protein genes were decreased by excessive light treatments (Alboresi *et al*. 2011). In the present study, the ultraviolet-B receptor UVR8-like gene, which has been reported to be involved in UV-protective responses (Rizzini *et al*. 2011), was also highly expressed in FSs (logFC: 6.294977, padj: 3.18E-06; data not shown). Likewise, artificial UV-B irradiation increased the expression of UV-B signalling genes including UVR8-like genes in the Antarctic moss *Leptobryum pyriforme* (Liu *et al*. 2021). These gene expression responses might be one of the common mechanisms of tolerance to light stresses and excessive UV exposure under midnight sun in Antarctica.

A number of PCESR genes have been reported to be related to early responses to environmental stress (Hahn *et al*. 2013). In the present study, however, the expression of only a few PCESR genes was upregulated in FSs (see Supporting Information Table S4). In *B*. *pseudotriquetrum* grown in Antarctica, stress tolerances appear to have already been established by altering their secondary metabolism such as lipid accumulation and alteration of fatty acid composition.

The present study is the first one showing actual gene expression profiles in mosses grown under Antarctic field conditions. The study also showed a possibility that lipid accumulation and change in fatty acid composition are major mechanisms for acquiring environmental stress tolerance in Antarctic mosses. Lu *et al*. (2022) have reported that the amount of storage lipids decreases in *B*. *pseudotriquetrum* treated with cold stress despite of an increase in the proportion of unsaturated fatty acids. In the present study, since the gene expression of oil body membrane proteins, such as oleosin, was much higher in FSs, we expected that the amount of storage lipids would be larger under Antarctic field conditions. These differences might be attributed to differences between the Antarctic environment and artificial stress treatments. In natural fields, plants are exposed to complex environmental factors for a long time. Thus, on-site analysis is essential for elucidating actual plant environmental responses and stress tolerance mechanisms.

## Supporting Information

The following additional information is available in the online version of this article –

**Table S1.** Location and environmental conditions of each sampling spot.

**Table S2.** List of primers used in the present study.

**Table S3.** Length statistics and composition of the assembled transcripts.

**Table S4.** Expression changes of the 56 PCESR genes involved in response for environmental stress in FSs compared with CSs. The responsiveness of each gene to oxidative, genotoxic, osmotic, high salinity, UV-B, and wounding stresses are shown in ‘○’.

**Figure S1.** Location of four sampling spots (spots 1–4). Photographs show views of each spot and corrected mosses. Map data were obtained Geospatial Information Authority of Japan (https://www.gsi.go.jp/antarctic/) and maps were created using QGIS3 software.

**Figure S2.** Flow of sample preparation and transcriptome analysis.

**Figure S3.** Representative significantly enriched photosynthesis-related KEGG (Kyoto Encyclopaedia of Genes and Genomes) pathways. Green boxes indicate significantly downregulated genes in FSs compared with CSs.

plae043_suppl_Supplementary_Material

## Data Availability

The sequencing data used in this study are openly available in the DDBJ Sequence Read Archive (DRA) with the accession number DRA016751.

## References

[CIT0001] Alboresi A , Dall’osto L, Aprile A, Carillo P, Roncaglia E, Cattivelli L, Bassi R. 2011. Reactive oxygen species and transcript analysis upon excess light treatment in wild-type *Arabidopsis thaliana* vs a photosensitive mutant lacking zeaxanthin and lutein. BMC Plant Biology 11:62. 21481232 10.1186/1471-2229-11-62 PMC3083342

[CIT0002] Aro EM , Karunen P. 1988. Effects of hardening and freezing stress on membrane lipids and CO2 fixation of *Ceratodon purpureus* protonemata. Physiologia Plantarum 74:45–52.

[CIT0003] Barrero-Sicilia C , Silvestre S, Haslam RP, Michaelson LV. 2017. Lipid remodelling: unravelling the response to cold stress in *Arabidopsis* and its extremophile relative *Eutrema salsugineum*. Plant Science : an international journal of experimental plant biology 263:194–200. 28818375 10.1016/j.plantsci.2017.07.017 PMC5567406

[CIT0004] Beike AK , Jaeger C, Zink F, Decker EL, Reski R. 2014. High contents of very long-chain polyunsaturated fatty acids in different moss species. Plant Cell Reports 33:245–254. 24170342 10.1007/s00299-013-1525-z PMC3909245

[CIT0005] Beike AK , Lang D, Zimmer AD, Wüst F, Trautmann D, Wiedemann G, Beyer P, Decker EL, Reski R. 2015. Insights from the cold transcriptome of *Physcomitrella patens*: global specialization pattern of conserved transcriptional regulators and identification of orphan genes involved in cold acclimation. The New Phytologist 205:869–881. 25209349 10.1111/nph.13004 PMC4301180

[CIT0006] Bhatla SC , Kaushik V, Yadav MK. 2010. Use of oil bodies and oleosins in recombinant protein production and other biotechnological applications. Biotechnology Advances 28:293–300. 20067829 10.1016/j.biotechadv.2010.01.001

[CIT0007] Cameron KD , Teece MA, Smart LB. 2006. Increased accumulation of cuticular wax and expression of lipid transfer protein in response to periodic drying events in leaves of tree tobacco. Plant Physiology 140:176–183. 16361524 10.1104/pp.105.069724 PMC1326042

[CIT0008] Carvalho AO , Gomes VM. 2007. Role of plant lipid transfer proteins in plant cell physiology-a concise review. Peptides 28:1144–1153. 17418913 10.1016/j.peptides.2007.03.004

[CIT0009] Degenkolbe T , Giavalisco P, Zuther E, Seiwert B, Hincha DK, Willmitzer L. 2012. Differential remodeling of the lipidome during cold acclimation in natural accessions of *Arabidopsis thaliana*. The Plant Journal: for Cell and Molecular Biology 72:972–982. 23061922 10.1111/tpj.12007

[CIT0010] Fan J , Yan C, Zhang X, Xu C. 2013. Dual role for phospholipid: diacylglycerol acyltransferase: enhancing fatty acid synthesis and diverting fatty acids from membrane lipids to triacylglycerol in *Arabidopsis* leaves. Plant Cell 25:3506–3518. 24076979 10.1105/tpc.113.117358 PMC3809546

[CIT0011] Fang S , Li T, Zhang P, Liu C, Cong B, Liu S. 2022. Integrated transcriptome and metabolome analyses reveal the adaptation of Antarctic moss *Pohlia nutans* to drought stress. Frontiers in Plant Science 13:924162. 36035699 10.3389/fpls.2022.924162 PMC9403716

[CIT0012] Gangadhar BH , Sajeesh K, Venkatesh J, Baskar V, Abhinandan K, Yu JW, Prasad R, Mishra RK. 2016. Enhanced tolerance of transgenic potato plants over-expressing non-specific lipid transfer protein-1 (*StnsLTP1*) against multiple abiotic stresses. Frontiers in Plant Science 7:1228. 27597854 10.3389/fpls.2016.01228 PMC4993012

[CIT0013] Guo L , Yang H, Zhang X, Yang S. 2013. *Lipid transfer protein 3* as a target of MYB96 mediates freezing and drought stress in *Arabidopsis*. Journal of Experimental Botany 64:1755–1767. 23404903 10.1093/jxb/ert040 PMC3617838

[CIT0014] Hahn A , Kilian J, Mohrholz A, Ladwig F, Peschke F, Dautel R, Harter K, Berendzen KW, Wanke D. 2013. Plant core environmental stress response genes are systemically coordinated during abiotic stresses. International Journal of Molecular Sciences 14:7617–7641. 23567274 10.3390/ijms14047617 PMC3645707

[CIT0015] Hart AJ , Ginzburg S, Xu M, Fisher CR, Rahmatpour N, Mitton JB, Paul R, Wegrzyn JL. 2020. EnTAP: bringing faster and smarter functional annotation to non-model eukaryotic transcriptomes. Molecular Ecology Resources 20:591–604. 31628884 10.1111/1755-0998.13106

[CIT0016] He M , He CQ, Ding NZ. 2018. Abiotic Stresses: General defenses of land plants and chances for engineering multistress tolerance. Frontiers in Plant Science 9:1771. 30581446 10.3389/fpls.2018.01771 PMC6292871

[CIT0017] Hrmova M , Hussain SS. 2021. Plant transcription factors involved in drought and associated stresses. International Journal of Molecular Sciences 22:5662. 34073446 10.3390/ijms22115662 PMC8199153

[CIT0018] Kim EY , Park KY, Seo YS, Kim WT. 2016. *Arabidopsis* small rubber particle protein homolog SRPs play dual roles as positive factors for tissue growth and development and in drought stress responses. Plant Physiology 170:2494–2510. 26903535 10.1104/pp.16.00165 PMC4825120

[CIT0019] Kopylova E , Noé L, Touzet H. 2012. SortMeRNA: fast and accurate filtering of ribosomal RNAs in metatranscriptomic data. Bioinformatics 28:3211–3217. 23071270 10.1093/bioinformatics/bts611

[CIT0020] Laibach N , Schmidl S, Müller B, Bergmann M, Prüfer D, Schulze Gronover C. 2018. Small rubber particle proteins from *Taraxacum brevicorniculatum* promote stress tolerance and influence the size and distribution of lipid droplets and artificial poly(cis-1,4-isoprene) bodies. The Plant Journal 93:1045–1061. 29377321 10.1111/tpj.13829

[CIT0021] Lata C , Prasad M. 2011. Role of DREBs in regulation of abiotic stress responses in plants. Journal of Experimental Botany 62:4731–4748. 21737415 10.1093/jxb/err210

[CIT0022] Liu WX , Liu HL, Qu LQ. 2013. Embryo-specific expression of soybean oleosin altered oil body morphogenesis and increased lipid content in transgenic rice seeds. TAG. Theoretical and Applied Genetics. Theoretische und Angewandte Genetik 126:2289–2297. 23748707 10.1007/s00122-013-2135-4

[CIT0023] Liu J , Yu T, Mu Z, Li G. 2019. TransLiG: a de novo transcriptome assembler that uses line graph iteration. Genome Biology 20:81. 31014374 10.1186/s13059-019-1690-7 PMC6480747

[CIT0024] Liu S , Fang S, Liu C, Zhao L, Cong B, Zhang Z. 2021. Transcriptomics integrated with metabolomics reveal the effects of ultraviolet-B radiation on flavonoid biosynthesis in Antarctic moss. Frontiers in Plant Science 12:788377. 34956286 10.3389/fpls.2021.788377 PMC8692278

[CIT0025] Liu S , Li T, Fang S, Zhang P, Yi D, Cong B, Zhang Z, Zhao L. 2022. Metabolic profiling and gene expression analysis provide insights into cold adaptation of an Antarctic moss *pohlia nutans*. Frontiers in Plant Science 13:1006991. 36176693 10.3389/fpls.2022.1006991 PMC9514047

[CIT0026] Livak KJ , Schmittgen TD. 2001. Analysis of relative gene expression data using real-time quantitative PCR and the 2(-Delta Delta C(T)) method. Methods 25:402–408. 11846609 10.1006/meth.2001.1262

[CIT0027] Love MI , Huber W, Anders S. 2014. Moderated estimation of fold change and dispersion for RNA-seq data with DESeq2. Genome Biology 15:550. 25516281 10.1186/s13059-014-0550-8 PMC4302049

[CIT0028] Lu Y , Eiriksson FF, Thorsteinsdóttir M, Simonsen HT. 2022. Lipidomic analysis of moss species *Bryum pseudotriquetrum* and *Physcomitrium patens* under cold stress. Plant-environment interactions (Hoboken, N.J.) 3:254–263. 37284430 10.1002/pei3.10095 PMC10168071

[CIT0029] Martín-Pedraza L , González M, Gómez F, Blanca-López N, Garrido-Arandia M, Rodríguez R, Torres MJ, Blanca M, Villalba M, Mayorga C. 2016. Two nonspecific lipid transfer proteins (nsLTPs) from tomato seeds are associated to severe symptoms of tomato-allergic patients. Molecular Nutrition & Food Research 60:1172–1182. 26840232 10.1002/mnfr.201500782

[CIT0030] Mikami K , Murata N. 2003. Membrane fluidity and the perception of environmental signals in cyanobacteria and plants. Progress in Lipid Research 42:527–543. 14559070 10.1016/s0163-7827(03)00036-5

[CIT0031] Nishiyama T , Hiwatashi Y, Sakakibara K, Kato M, Hasebe M. 2000. Tagged mutagenesis and gene-trap in the moss, *Physcomitrella patens* by shuttle mutagenesis. DNA Research 7:9–17. 10718194 10.1093/dnares/7.1.9

[CIT0032] Patro R , Duggal G, Love M, Irizarry RA, Kingsford C. 2017. Salmon provides fast and bias-aware quantification of transcript expression. Nature Methods 14:417–419. 28263959 10.1038/nmeth.4197 PMC5600148

[CIT0033] Pérez-Rubio P , Lottaz C, Engelmann JC. 2019. FastqPuri: high-performance preprocessing of RNA-seq data. BMC Bioinformatics 20:226. 31053060 10.1186/s12859-019-2799-0 PMC6500068

[CIT0034] Resemann HC , Herrfurth C, Feussner K, Hornung E, Ostendorf AK, Gömann J, Mittag J, van Gessel N, Vries J, Ludwig-Müller J, et al. 2021. Convergence of sphingolipid desaturation across over 500 million years of plant evolution. Nature Plants 7:219–232. 33495556 10.1038/s41477-020-00844-3

[CIT0035] Reszczyńska E , Hanaka A. 2020. Lipids composition in plant membranes. Cell Biochemistry and Biophysics 78:401–414. 33034870 10.1007/s12013-020-00947-w PMC7567678

[CIT0036] Rizzini L , Favory JJ, Cloix C, Faggionato D, O’Hara A, Kaiserli E, Baumeister R, Schäfer E, Nagy F, Jenkins GI, et al. 2011. Perception of UV-B by the *Arabidopsis* UVR8 protein. Science 332:103–106. 21454788 10.1126/science.1200660

[CIT0037] Rochaix JD , Bassi R. 2019. LHC-like proteins involved in stress responses and biogenesis/repair of the photosynthetic apparatus. The Biochemical Journal 476:581–593. 30765616 10.1042/BCJ20180718

[CIT0038] Shimada TL , Shimada T, Takahashi H, Fukao Y, Hara-Nishimura I. 2008. A novel role for oleosins in freezing tolerance of oilseeds in *Arabidopsis thaliana*. The Plant Journal: for Cell and Molecular Biology 55:798–809. 18485063 10.1111/j.1365-313X.2008.03553.x

[CIT0039] Soneson C , Love MI, Robinson MD. 2015. Differential analyses for RNA-seq: transcript-level estimates improve gene-level inferences. F1000Research 4:1521–1578. 26925227 10.12688/f1000research.7563.1 PMC4712774

[CIT0040] Sterk P , Booij H, Schellekens GA, Van Kammen A, De Vries SC. 1991. Cell-specific expression of the carrot EP2 lipid transfer protein gene. Plant Cell 3:907–921. 1822991 10.1105/tpc.3.9.907 PMC160059

[CIT0041] Ting JT , Lee K, Ratnayake C, Platt KA, Balsamo RA, Huang AH. 1996. Oleosin genes in maize kernels having diverse oil contents are constitutively expressed independent of oil contents. Planta 199:158–165. 8680304 10.1007/BF00196892

[CIT0042] Tzen JTC , Cao YZ, Laurent P, Ratnayake C, Huang AHC. 1993. Lipids, proteins, and structure of seed oil bodies from diverse species. Plant Physiology 101:267–276. 12231682 10.1104/pp.101.1.267 PMC158673

[CIT0043] Vanhercke T , El Tahchy A, Liu Q, Zhou XR, Shrestha P, Divi UK, Ral JP, Mansour MP, Nichols PD, James CN, et al. 2014. Metabolic engineering of biomass for high energy density: oilseed-like triacylglycerol yields from plant leaves. Plant Biotechnology Journal 12:231–239. 24151938 10.1111/pbi.12131 PMC4285938

[CIT0044] Xu Y , Zheng X, Song Y, Zhu L, Yu Z, Gan L, Zhou S, Liu H, Wen F, Zhu C. 2018. NtLTP4, a lipid transfer protein that enhances salt and drought stresses tolerance in *Nicotiana tabacum*. Scientific Reports 8:8873. 29891874 10.1038/s41598-018-27274-8 PMC5995848

[CIT0045] Yu C , Wang HS, Yang S, Tang XF, Duan M, Meng QW. 2009. Overexpression of endoplasmic reticulum omega-3 fatty acid desaturase gene improves chilling tolerance in tomato. Plant Physiology and Biochemistry : PPB 47:1102–1112. 19648018 10.1016/j.plaphy.2009.07.008

[CIT0046] Zhang J , Liu H, Sun J, Li B, Zhu Q, Chen S, Zhang H. 2012. Arabidopsis fatty acid desaturase FAD2 is required for salt tolerance during seed germination and early seedling growth. PLoS One 7:e30355. 22279586 10.1371/journal.pone.0030355 PMC3261201

[CIT0047] Zhang W , Liu S, Li C, Zhang P, Zhang P. 2019. Transcriptome sequencing of Antarctic moss under salt stress emphasizes the important roles of the ROS-scavenging system. Gene 696:122–134. 30790651 10.1016/j.gene.2019.02.037

