# Supplementary material for: Transcriptome analysis of the common moss Bryum pseudotriquetrum grown under Antarctic field condition
Source: AoB Plants. 2024 Aug 10;16(5):plae043. doi: 10.1093/aobpla/plae043 (PMC11430918; doi:10.1093/aobpla/plae043)
Supplement: plae043_suppl_Supplementary_Material [file plae043_suppl_supplementary_material.zip › Supporting Information.docx]

**Supporting information**

**Table S1. Location and environmental conditions of each sampling spot.**

| **Sampling spot** | **Location** | **Conditions** |
| --- | --- | --- |
| **Spot 1** | Langhovde  S 69°14’52.2”, E 39°43’09.3” | Green moss mats (3–4 cm in thickness) developed on sandy soil nearby a freshwater lake. |
| **Spot 2** | Skarvsnes  S 69°28’50.3”, E 39°38’54.3” | Partially blackish and dry moss mats (5–6 cm in depth) developed on sandy and saline soil nearby Suribachi-Ike (salt lake). |
| **Spot 3** | Skarvsnes  S 69°29’34.4”, E 39°34’44.4” | Green and wet moss mats (4–5 cm in depth) developed on rock nearby Nogiku-Ike (freshwater lake). |
| **Spot 4** | Skallen  S 69°40’46.1”, E 39°29’19.7” | Light green and very wet moss mats (6–8 cm in depth) developed on sandy soil along by a stream of fresh water. |

**Table S2. List of primers used in the present study.**

| **Primer name** | **Sequence (5’→3’)** | **PCR products** | **Contig ID of target gene** | **Note** |
| --- | --- | --- | --- | --- |
| qRT-942-Fw | GAGTCTACTCTCAAGCTTTCCACTTG | 169 bp | Graph_942 | oleosin Bn-III |
| qRT-942-Rev | AAGTGGGCTTCATTCTTGCTG |  |  |  |
| qRT-1034-Fw | GAGGTATGAACCAGTGGCTGAG | 184 bp | Graph_1034 | REF/SRPP |
| qRT-1034-Rev | CAACGGGATGTAGTAAGCCAAG |  |  |  |
| qRT-8732-Fw | GCAGCTTCAGCAACTCCAGAAC | 179 bp | Graph_8732 | Δ15 fatty acid desaturase, endoplasmic reticulum (Δ15FAD; FAD3) |
| qRT-8732-Rev | CGTGAGCGTTTTGTAGAGACCTTC |  |  |  |
| qRT-1415-Fw | GGCTTGCGTTGCCAGTTATC | 142 bp | Graph_1415 | Δ15 fatty acid desaturase, endoplasmic reticulum (Δ15FAD; FAD3) |
| qRT-1415-Rev | GTTGTTTACCGTCTTACTGCTGGAG |  |  |  |
| qRT-3920-Fw | GATTCCGTTGCTGATTGTGAAC | 142 bp | Graph_3920 | Δ12 fatty acid desaturase, endoplasmic reticulum (Δ12FAD; FAD2) |
| qRT-3920-Rev | CACTCCATAGTCTCGGTCCATG |  |  |  |
| qRT-1605-Fw | ATCTCATCGAGGATTCCAAGCTAC | 142 bp | Graph_1605 | Δ12 fatty acid desaturase, chloroplast (Δ12FAD; FAD6) |
| qRT-1605-Rev | TATCGTCGAATAGATTGCATTGTG |  |  |  |
| qRT-4287-Fw | ACTGTGCTTGGACACTATGCTTG | 189 bp | 4287 | acyl-lipid (9-3)-desaturase (Δ8FAD; FADS2) |
| qRT-4287-Rev | AATCTGGGCATTGACGAAGTC |  |  |  |
| qRT-6000-Fw | CGACAAGGATGTGCAAGCAAC | 185 bp | 6000 | lysophospholipid acyltransferase 1 (LPCAT1) |
| qRT-6000-Rev | GAGTTATCAGGAGCTGTGGGAATC |  |  |  |
| qRT-4090-Fw | TCAACATTAAGGACTTTGGAGACAG | 117 bp | Graph_4090 | phosphatidate cytidylyltransferase 1 (CDS1) |
| qRT-4090-Rev | GAGCAATGAAAGACTGGTAATAGATG |  |  |  |
| qRT-1342-Fw | TGTTGGCGCTGTTCTACATTC | 184 bp | Graph_1342 | linoleate 9S-lipoxygenase (9-LOX) |
| qRT-1342-Rev | CCATGTTTGGCGTACAAGTTCTC |  |  |  |
| qRT-3502-Fw | ATGCCCTGAAGTGTTCGAGAAG | 103 bp | Graph_3502 | cytochrome b5 |
| qRT-3502-Rev | GACCTTACTCAAGATGCCAACAG |  |  |  |
| qRT-9286-Fw | AAGGACTACGATTGCACGAACTC | 126 bp | Graph_9286 | non-specific lipid transfer protein (ns-LTP) |
| qRT-9286-Rev | TTCCTTCGCTTCAGTGGTGAC |  |  |  |
| qRT-3730-Fw | GTGTGATGGTGGTGGTGGTAG | 176 bp | Graph_3730 | non-specific lipid-transfer protein 2B (ns-LTP 2B) |
| qRT-3730-Rev | CCCATACTGAGCAAACGCAG |  |  |  |
| qRT-BpPOB1-Fw | GAGTCTACTCTCAAGCTTTCCACTTG | 174 bp | - | BTB/POZ domain-containing protein |
| qRT-BpPOB1-Rev | AAGTGGGCTTCATTCTTGCTG |  |  |  |

**Table S3. Number of reads and read mapping rates in each sample.**

| Sample | Number of total reads | Number of mapped reads | Mapping rate^a^ |
| --- | --- | --- | --- |
| CS1 | 4,020,365 | 3,718,816 | 92.5 % |
| CS2 | 4,738,586 | 4,347,384 | 91.7 % |
| CS3 | 3,134,874 | 2,913,542 | 92.9 % |
| FS1 | 3,450,372 | 2,972,577 | 86.2 % |
| FS2 | 3,305,153 | 2,566,026 | 77.6 % |
| FS3 | 3,744,192 | 2,977,342 | 79.5 % |

^a^ Mapping rate indicates the percentage of mapped reads relative to the total number of reads.

**Table S4. Length statistics and composition of the assembled transcripts.**

| Number of sequences | 88,205 |
| --- | --- |
| Total length (nt) | 140,334,145 |
| Longest sequence (nt) | 39,314 |
| Shortest sequence (nt) | 200 |
| Mean sequence length (nt) | 1,591 |
| Median sequence length (nt) | 1,231 |
| N50 sequence length (nt) | 2,250 |
| L50 sequence count | 19,901 |
| Base composition (%) | A:25.03  T:26.82  G:25.91  C:22.24  N: 0.00  Other: 0.00 |
| GC-content (%) | 48.15 |


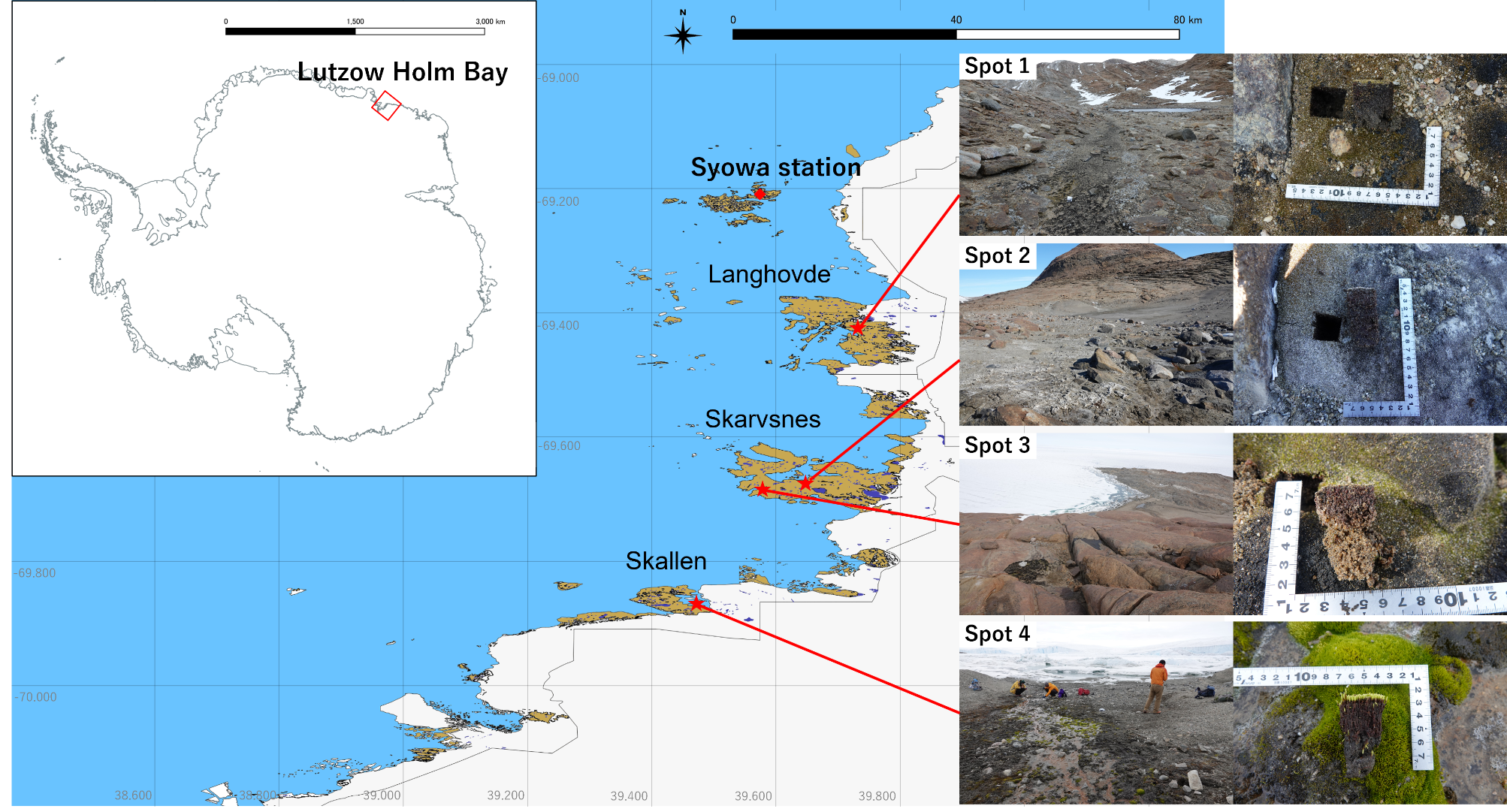


**Figure S1. Location of four sampling spots (Spot 1–4).** Photographs show views of each spot and corrected mosses. Map data were obtained Geospatial Information Authority of Japan (https://www.gsi.go.jp/antarctic/) and map were created using QGIS3 software.

**
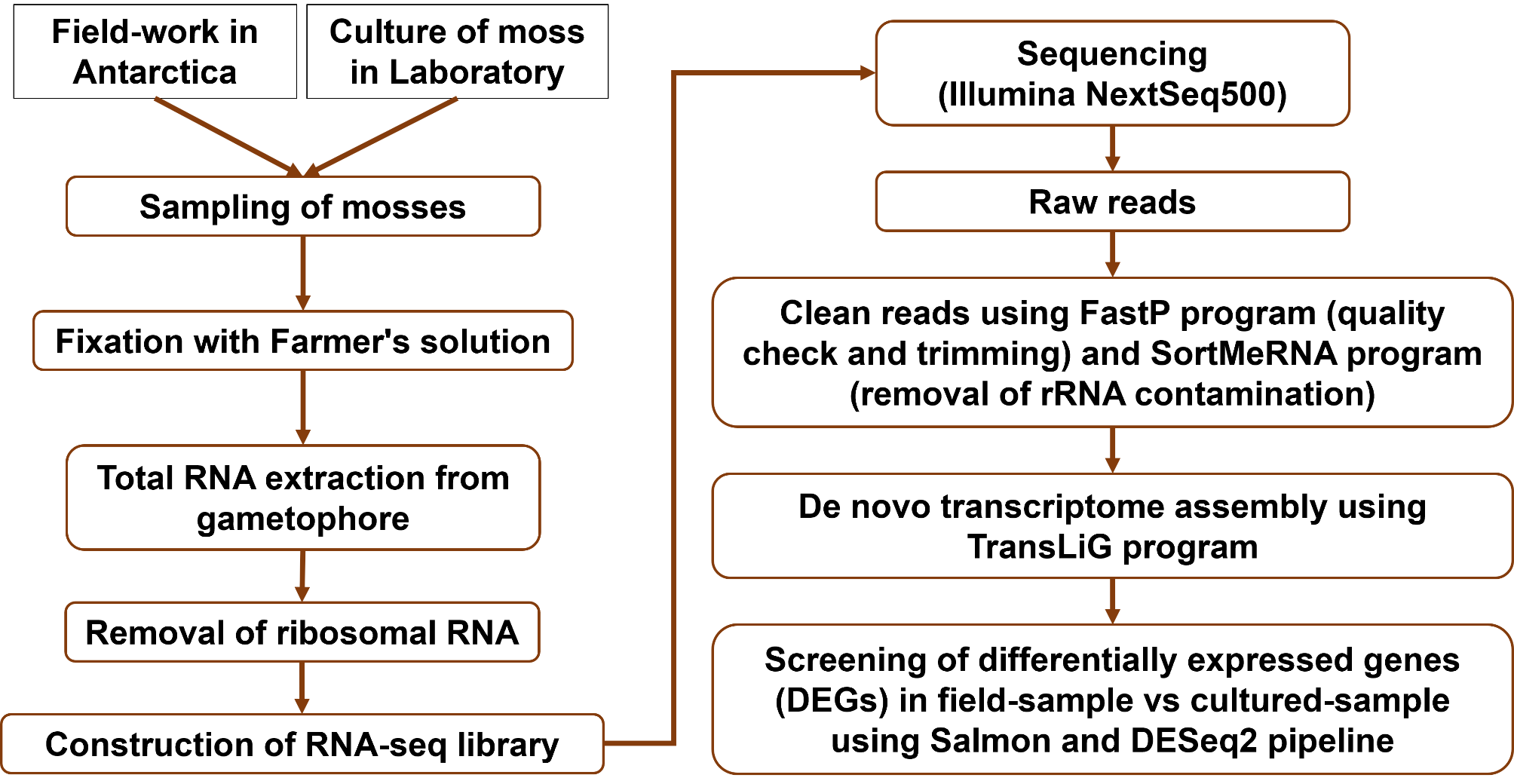
**

**Figure S2. Flow of sample preparation and transcriptome analysis.**


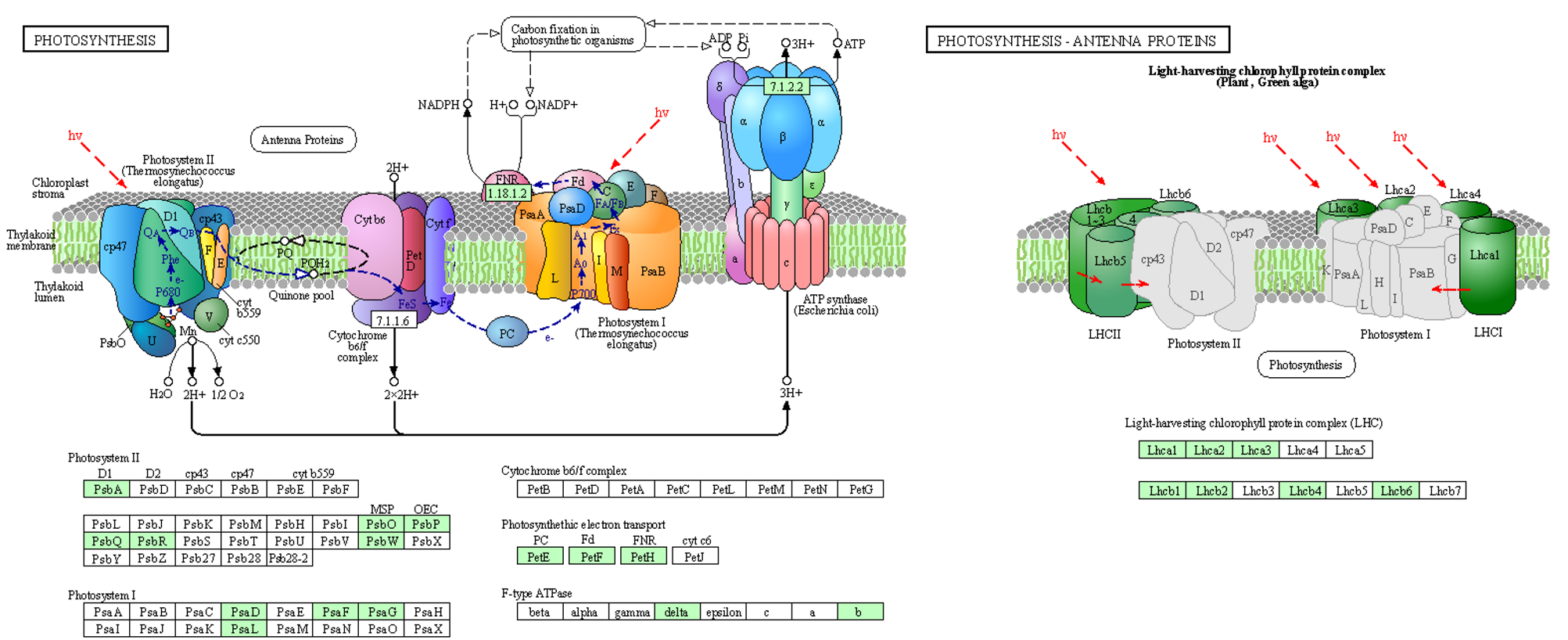


**Figure S3. Representative significantly enriched photosynthesis-related KEGG (Kyoto Encyclopedia of Genes and Genomes) pathways.**
